# Development of a Lyophilized Off-the-Shelf Mesenchymal Stem Cell-Derived Acellular Therapeutic

**DOI:** 10.3390/pharmaceutics14040849

**Published:** 2022-04-13

**Authors:** Julia Driscoll, Irene K. Yan, Tushar Patel

**Affiliations:** Department of Transplantation, Mayo Clinic, Jacksonville, FL 32224, USA; driscoll.julia@mayo.edu (J.D.); yan.irene@mayo.edu (I.K.Y.)

**Keywords:** secretome, extracellular vesicles (EV), acellular therapeutic, lyopreservation

## Abstract

The therapeutic activities elicited by mesenchymal stem cells (MSC) are in part mediated through paracrine action by the release of extracellular vesicles (EV) and secreted proteins. Collectively, these MSC-derived factors, referred to as the secretome product (SP), are intrinsically therapeutic and represent an attractive alternative to cell-based therapies. Herein, we developed a lyopreservation protocol to extend the shelf-life of the MSC-SP without compromising the structural or functional integrity of the vesicular components. The SP isolated from normoxia- and anoxia-exposed MSC elicited protective effects in an in vitro model of oxidative injury and the bioactivity was retained in the lyophilized samples. Three separate formulations of MSC-SP were isolated by tangential flow filtration using sucrose, trehalose, and mannitol as lyoprotectant agents. The MSC-SPs were lyophilized using a manifold protocol and the structural and functional integrity were assessed. The trehalose formulation of SP exhibited the highest EV and protein recovery after manifold-based lyophilization. To facilitate development as a therapeutic, a shelf lyophilization protocol was developed which markedly enhanced the recovery of EV and proteins. In conclusion, lyophilization represents an efficient method to preserve the structural and functional integrity of the MSC-SP and can be used to develop an off-the-shelf therapeutic.

## 1. Introduction

Mesenchymal stem cells (MSC) are widely recognized for their therapeutic and regenerative activities. These cells have multipotent differentiation potential and can be harvested from various postnatal tissues, including bone marrow, adipose tissue, and umbilical cord blood [1,2,3]. MSC possess several intrinsic therapeutic properties and have been reported to promote tissue regeneration, modulate immune responses, stimulate angiogenesis, and mitigate fibrosis [4,5,6,7,8]. Due to their wide-ranging therapeutic properties, the efficacy of MSC-based therapeutics has been explored in numerous clinical trials [9,10,11]. While MSC-based therapeutics have shown promising potential, the low retention and engraftment rates of these cells in vivo suggest that MSC mediate their therapeutic effects through paracrine action, via the release of extracellular vesicles (EV) and secretion of soluble proteins [12,13].

These MSC-derived EV and soluble proteins, collectively referred to as the *secretome product* (SP), retain many of the intrinsic therapeutic properties of their parental cells of origin [14,15]. Mesenchymal stem cell-derived EV have been shown to contain a diverse array of bioactive cargo, including proteins, nucleic acids, non-coding RNA and lipids. The lipid bilayer of the EV membrane protects the cargo from degradation and is often endowed with certain receptors, surface proteins and/or lipids that enable the targeted delivery of the cargo to the desired location [16]. The soluble protein fraction of the secretome has been reported to include angiogenic and growth factors, extracellular matrix remodeling proteins, and various immune modulators, including cytokines and chemokines, to name a few [17,18]. The MSC-SP preferentially homes to sites of inflammation or injury [19,20]. The composition of the secretome can easily be modified to enrich factors of EV cargo and/or to modify the targeting abilities [21].

The MSC-SP is an attractive alternative to MSC cell-based therapeutics for several reasons. The MSC-secretome has a better safety profile, due to the acellular nature of the material; namely, it is less immunogenic, and has negligible tumorigenic potential [22,23]. The MSC-secretome also overcomes many of the challenges associated with the clinical implementation of cell-based therapeutics. Indeed, the secretome product can be isolated using scalable, good manufacturing practice (GMP)-compliant production platforms and is cost efficient to develop and store. For these reasons, efforts are underway to develop MSC-secretome therapeutics for clinical applications. To ensure the successful implementation of secretome therapeutics in the clinic, storage conditions need to be optimized. At present, several cryopreservation techniques have been investigated with varying degrees of success [24,25,26]. The challenges with cryopreservation include low recovery yields, compromised EV membrane integrity, and attenuation of the EV/SP-mediated functional activities, although EV can be stored for several months at −80 °C without any negative impact on its structure or bioactivity [25,27].

Herein, we sought to extend the biological half-life of the MSC-SP towards the creation of a ready-to-use, off-the-shelf product. Using tangential flow filtration, three batches of MSC-SP, each diafiltrated with a different sugar-based formulation, were isolated and tested. A shelf-based lyophilization protocol was then developed to create a freeze-dried MSC-SP with an extended shelf-life, and the effects on structural properties and the biological activity were assessed after recovery.

## 2. Materials and Methods

### 2.1. Cells and Cell Culture

Primary human bone marrow-derived mesenchymal stem cells (MSC, Lonza, Basel, Switzerland) were cultured in MSC basal media supplemented with 10% fetal bovine serum (FBS), 2% l-glutamine and 0.1% gentamicin (Lonza, Basel, Switzerland). HepG2 hepatocarcinoma cells (ATCC, Manassas, VA, USA) were cultured on collagen-coated plates in Dulbecco’s modified eagle medium (DMEM, Fisher Scientific, Waltham, MA, USA) supplemented with 10% FBS (Gemini Bio Products, Sacramento, CA, USA) and 1% penicillin-streptomycin.

### 2.2. Generation of Vesicle Depleted Media

Five hundred milliliters of MSC was transferred to a 500 mL reservoir and loaded into the kR2i Kross Flow Tangential Flow Filtration (TFF) system furnished with a modified polyethylersulfane (mPES)-coated D02-E010-05-S hollow fiber filter (Spectrum Labs, Los Angeles, CA, USA). The TFF system was set to run on manual mode at a flow speed of 30 mL/min. The permeate was collected and subsequently passed through a sterile 0.22 µM filter and stored at 4 °C for future use.

### 2.3. Anoxia Conditioning of MSC

Passage three MSC were cultured in vesicle-depleted complete media for 24 h. The MSC were then subjected to anoxia (5% CO_2_/95% N_2_ for 24 h (aMSC)) or kept under normoxic conditions for an additional 24 h (nMSC). MSC-conditioned media (MSC-CM) were then collected and stored at 4 °C.

### 2.4. Isolation of MSC-Secretome Product (MSC-SP)

The MSC-CM underwent sequential centrifugation at 300× *g* for 5 min, followed by a spin at 2000× *g* for 30 min at 4 °C to remove cell and cellular debris contaminants. The supernatant was loaded into the kR2i TFF system equipped with a D02-E010-05-S filter, with a molecular weight cut-off of 10 kDa (Spectrum Labs, Los Angeles, CA, USA). The flow rate was maintained at 30 mL/min and the shear rate did not exceed 2000 s^−1^. The product was concentrated to reduce the volume to 5 mL, and then diafiltrated five times with either PBS, 5% *w*/*v* sucrose, 4% *w/v* mannitol or 4% *w*/*v* trehalose. The material was further concentrated two times to achieve a final volume of 3 mL. The isolated MSC-secretome products (MSC-SP) were stored at 4 °C. The filter was washed with PBS between consecutive isolations.

### 2.5. Manifold-Based Lyophilization of MSC-SP

Following isolation by TFF, the resulting MSC-SP samples were separated into equal volume aliquots and one aliquot of each sample was stored at 4 °C and the others were stored overnight in a cool cell at −80 °C. The frozen MSC-SP samples were lyophilized for 48 h using a freeze FreeZone Plus 6, which was maintained at 3 × 10^−3^ mBAR and −81 °C (Labcono, Kansas City, MO, USA). The resulting lyophilized MSC-SP samples were reconstituted in 100 µL of the appropriate lyoprotectant agent. The samples were evaluated by BCA assay and NTA analysis to assess the protein and particle recovery, respectively.

### 2.6. Reactive Oxygen Species (ROS) Assay

ROS levels were detected using 2′,7′-Dichlorodihydrofluorescein acetate (DCFDA). This is a cell-permeable probe that is non-fluorescent. Once taken up by cells, DCFDA is converted by intracellular esterases and oxidized by intracellular ROS to 2′,7′dichlorofluorescein, which is highly fluorescent. The increase in fluorescence is a sensitive indicator of the presence of ROS. HepG2 cells were seeded onto a black collagen-coated 96-well plate at a concentration of 10,000 cells/well. After an overnight attachment period, the cells were treated with 4% *w/v* trehalose (vehicle control), 8.00 × 10^6^ particles of fresh or 1.00 × 10^8^ particles of the lyophilized trehalose formulation of MSC-SP. After 24 h, the cells were incubated with 20 µM of DCFDA for 45 min in complete darkness at 37 °C. The media were then removed and replaced either with media or media supplemented with 0.5 mM tert-butyl hydrogen peroxide (TBHP) for 1 h. ROS specific production was quantitated by measuring the fluorescence intensity at an excitation wavelength of 485 nm and emission of 535 nm.

### 2.7. Shelf-Based Lyophilization of MSC-SP

A shelf-based lyophilization protocol using the VirTis AdVantage 2.0 (SP scientific, Warminster, PA, USA) was developed to improve the protein and EV recovery. In brief, fresh MSC-SP aliquot samples were subjected to thermal treatment in which the shelf temperature was gradually reduced to −40 °C for 150 min, while the pressure was maintained at 760 Torr. The pressure was subsequently reduced to 200 mTorr for 300 min and the shelf temperature was maintained at −40 °C. The primary drying phase was initiated by progressively increasing the shelf temperature to 20 °C over a 900-min period, while the pressure was kept at 200 mTorr (Table 1). The resulting lyophilized MSC-SP were stored at 4 °C. The lyophilized samples were reconstituted with the appropriate lyoprotectant equal in volume to that of the original aliquot. The samples were evaluated by BCA assay and NTA analysis to assess the protein and EV recovery, respectively.

### 2.8. Particle Concentration and Size Distribution

Nanoparticle tracking analysis of the MSC-SP samples was performed using a Nanosight LM10 (Malvern Pananalytical, Westborough, MA, USA). Three videos were captured, and the average size and particle concentration were quantified. The NTA 3.3 software was used for data analysis.

### 2.9. Protein Quantification

A BCA assay was performed to quantify the protein content of the samples according to the manufacturer’s protocol (ThermoFisher Scientific, Waltham, MA, USA). Briefly, 80 µL of the reagent A + B solution was added to each well containing the MSC-SP samples. The plate was incubated at 37 °C for 30 min in complete darkness and the absorbance was subsequently measured at 562 nm using a FLUOstar omega plate reader (BMG Labtech, Cary, NC, USA). A standard curve of the blank corrected data was generated using a four-parameter fit.

## 3. Results

### 3.1. Development of Freeze-Dried MSC-SP

**Figure 1:**
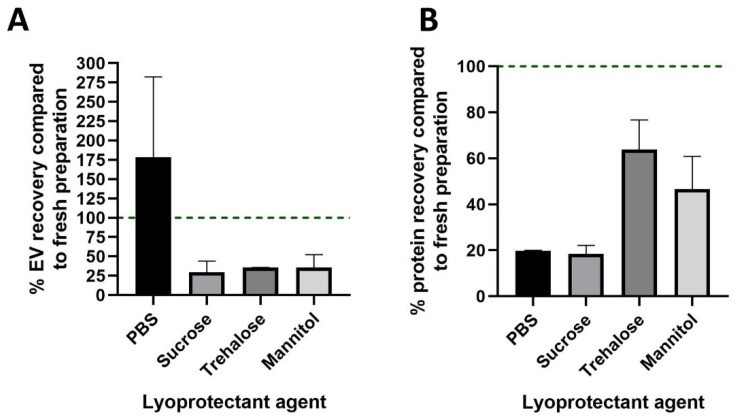
Characterization of the EV and protein recoveries in the manifold lyophilized MSC-SP. The secretome product (SP) was isolated from the MSC-conditioned media by tangential flow filtration (TFF). The diafiltrate used in TFF was altered to test four lyophilization excipients: PBS, 5% *w/v* sucrose, 4% *w/v* trehalose and 4% *w/v* mannitol. Lyophilized MSC-SPs were prepared using a manifold protocol and the EV and protein constituents were quantified by nanoparticle tracking analysis and BCA assay, respectively. The percentages of (**A**) EV and (**B**) protein recovered in the lyophilized MSC-SP were calculated.

To extend the shelf life of the MSC-SP, we developed a manifold-based protocol to prepare freeze-dried MSC-SPs. Lyoprotectant excipients were selected based on their ability to preserve EV and protein stability during lyophilization. The stability of nanoparticles such as EV is reported with the use of trehalose and mannitol (0.5–4% *w/v*) [28], as well as with 5% *w/v* sucrose [29]. Therefore, these sugar-based excipients were selected as potential lyoprotectant candidates. Four formulations of MSC-SP were prepared to assess the effectiveness of different lyoprotectant excipients: PBS, 5% *w/v* sucrose, 4% *w/v* trehalose and 4% *w/v* mannitol. Following the lyophilization of the MSC-SPs, the samples were reconstituted, and the EV and protein contents were quantified and compared to the respective fresh counterpart MSC-SP sample. The nanoparticle recovery in the PBS MSC-SP formulation exceeded 150%, which is suggestive of EV shearing (Figure 1A), whereas the EV recovery in the sugar formulations of MSC-SP was greater than 20% for all excipients tested. Next, we evaluated the protein recovery in the lyophilized MSC-SPs. The protein recovery was highest in the MSC-SP prepared in trehalose (Figure 1B). Therefore, the trehalose formulation of MSC-SP was chosen for subsequent functional assessments.

### 3.2. Lyophilized MSC-SP Retain Their Bioactivity

**Figure 2:**
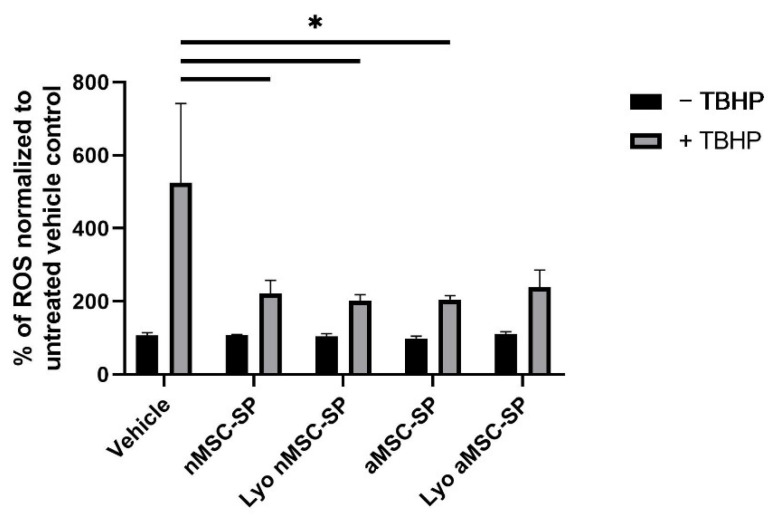
Antioxidant effects elicited by MSC-SP are maintained post-lyophilization. To alter the composition of the MSC-secretome product, MSC were exposed to normoxia or anoxia. The secretome products were isolated from the normoxic (nMSC-SP) or anoxic (aMSC-SP) preconditioned MSC by tangential flow filtration and separated into equal-volume aliquots. One aliquot of each sample was lyophilized using the manifold protocol (Lyo). HepG2 cells were pre-treated with 8.00 × 10^8^ particles of fresh or 1.00 × 10^10^ particles of lyo nMSC-SP or aMSC-SP for 24 hr. After this, half the cells in each treatment condition were exposed to 0.5 mM TBHP for 1 hr. A DCFDA assay was performed to quantify the levels of reactive oxygen species (ROS). (*) represents *p* < 0.05.

Previous research has shown that MSC are dynamic cells and alter their secretome profile to adapt to changing microenvironments [30]. To develop a SP with enhanced antioxidant effects, MSC were cultured under normoxic or anoxic conditions. The SP from normoxia (nMSC-SP) and anoxia (aMSC-SP) exposed cells were isolated and separated into equal-volume aliquots and one aliquot of each sample was subjected to lyophilization. Next, we examined whether the biological activity of MSC-SP was preserved in the lyophilized material. The therapeutic activity of fresh and lyophilized nMSC-SP and aMSC-SP was investigated using an in vitro model of oxidative injury. Pre-treatment with nMSC-SP or aMSC-SP significantly protected HepG2 cells from TBHP-induced oxidative injury (*p* < 0.05, Figure 2). However, there was no difference in the protective effects elicited by nMSC-SP and aMSC-SP. Therefore, the MSC-SP lyophilized using the manifold protocol retain their bioactivity.

### 3.3. Shelf-Based Lyophilization Improved EV and Protein Recovery

**Figure 3:**
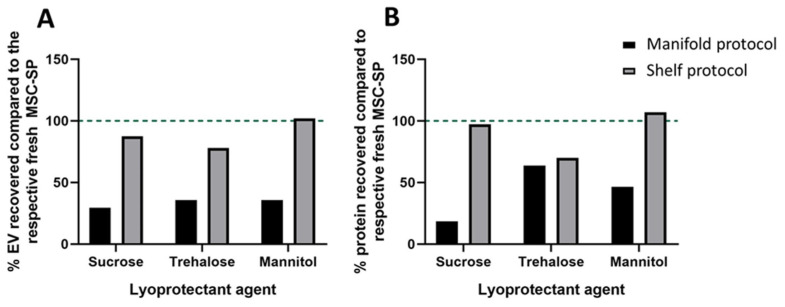
Comparison of the structural characteristics of freeze-dried MSC-SP prepared using two different lyophilization methods. The MSC-SP were isolated by tangential flow filtration using three different diafiltration agents, 5% *w/v* sucrose, 4% *w/v* trehalose and 4% *w/v* mannitol. The MSC-SP were subsequently separated into equal volume aliquots. Two aliquots of each sample were lyophilized using either a manifold- or a shelf-based protocol, and the third aliquot was stored at 4 °C. The lyophilized MSC-SP were reconstituted, and the EV and protein constituents were quantified by nanoparticle tracking analysis and BCA assay, respectively. The (**A**) EV and (**B**) protein recoveries in the lyophilized MSC-SP were calculated.

**Figure 4:**
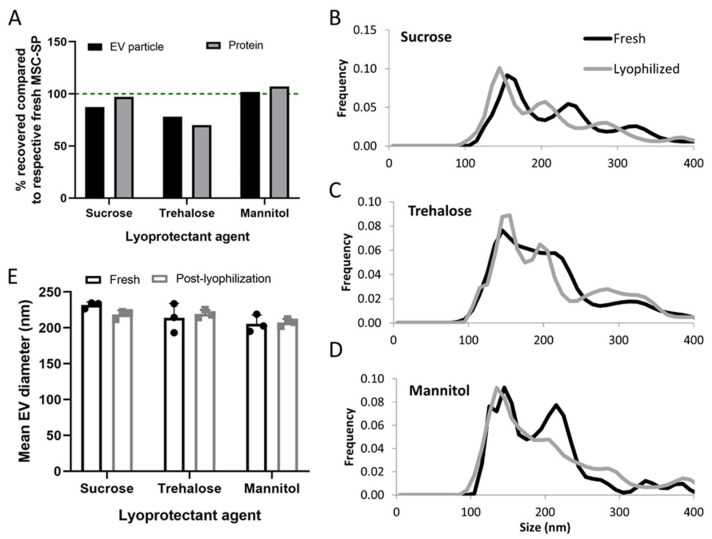
Characterization of the structural properties of MSC-SP lyophilized using the shelf-based protocol. MSC-SP were lyophilized using a shelf-based protocol and the EV and protein constituents were quantified by nanoparticle tracking analysis (NTA) and BCA assay, respectively. The (**A**) EV and protein recoveries of the lyophilized MSC-SP were calculated. (**B**–**D**) The size distribution profiles and the (**E**) mean diameters of the EV within the fresh and lyophilized MSC-SP were quantified using NTA.

With the advent of new lyophilization technology, we developed and tested a shelf-based lyophilization protocol specifically designed for the preservation of cell-derived trophic factors, including EV and secreted proteins. As before, we prepared three formulations of MSC-SP using lyophilization excipients, 5% sucrose, 4% *w/v* trehalose and 4% *w/v* mannitol. The SPs were separated into equal-volume aliquots and one aliquot of each formulation was freeze-dried using the newly developed shelf-based protocol. The MSC-SPs preserved using the shelf-based protocol had markedly higher EV and protein recoveries compared to those prepared with the manifold-based lyophilization protocol (Figure 3A,B). The EV and protein recoveries of the lyophilized MSC-SP were the highest in the samples diafiltrated with sucrose or mannitol (Figure 3A,B and Figure 4A). Differences in the particle size distribution profiles of the fresh and lyophilized secretome were observed; however, the average diameters of the EV were similar (Figure 4B–E; Table 2).

## 4. Discussion

MSC-EV and SP represent attractive therapeutics for a variety of reasons. These materials offer lower immunogenicity profiles, are relatively inexpensive to isolate compared to the costs associated with maintaining MSC and can be modified to enhance the therapeutic effectiveness and/or to ensure targeted delivery [22,31]. For these reasons, several clinical trials are already underway to investigate the therapeutic potential of the MSC-derived secretome and EV in a multitude of diseases and conditions. However, there are several obstacles that need to be addressed to ensure the successful translation to and implementation of these therapeutics in the clinical setting [22]. The MSC-SP must be isolated in compliance with GMP standards in a manner that is easily scalable to ensure adequate amounts of MSC-SP are available for clinical use [15]. Furthermore, the storage conditions of the MSC-SP must be optimized, so that the material can be stored for long periods of time without compromising the structural and functional integrity of the MSC-SP.

In the present study, we developed a method to generate an “off-the-shelf” MSC-derived acellular therapeutic to address some of the abovementioned issues. Herein, we utilized TFF to isolate the secretome product from bone marrow-derived MSC. We developed a manifold-based lyophilization protocol to prepare freeze-dried formulations of MSC-SP. The resulting lyophilized MSC-SP retained their bioactivity in vitro. Lastly, we developed an improved shelf-based lyophilization protocol that resulted in lyophilized MSC-SP with superior EV and protein recoveries, compared to those prepared using a manifold-based protocol. The MSC-SP lyophilized with the shelf protocol retained their structural integrity.

Compared to the traditional method of EV isolation, ultracentrifugation, TFF enables the isolation of both EV and secreted proteins in a GMP-compliant manner, which can be scaled up for increased production. The ability to process high volumes of MSC-CM addresses one of the challenges in developing MSC-SP-based therapeutics for clinical use. Extracellular vesicles isolated by this method retain their structural integrity, since they are not subjected to the high speeds required for ultracentrifugation-based EV isolation [32]. This feature of TFF-based EV isolation is especially appealing, given the important role the EV membrane has in protecting the intravesicular cargo and the roles that the EV surface proteins play in both homing and targeted delivery. Other attractive features of TFF-based EV isolation are that it can be set up to operate autonomously and can process a lower volume of starting material than that required for ultracentrifugation-based isolation [33]. In addition, TFF enables the isolation of secreted proteins in addition to EV. The soluble proteins present within the MSC-SP act in synergy with the EV to elicit enhanced therapeutic effects, compared to those elicited by treatment with the proteins or EV alone [34]. The availability of different molecular weight cut-off TFF filters allows users to tailor the size profile of the isolated EV and secreted proteins, thus enabling the isolation of an MSC-SP with the desired constituents.

A challenge in translating MSC-EV and MSC-SP based therapeutics to the clinic is the optimization of storage conditions to minimize the loss of material and to retain functional activity. Previous studies have shown that storage at −80 °C resulted in a minimal reduction in EV count; however, increased storage periods resulted in the formation of aggregates [35]. The EV stored under these conditions retained the surface expression of several hallmark tetraspannin markers and exhibited minimal loss of protein and RNA cargo [36]. However, the costs associated with long-term storage at this temperature limit its clinical utility [37]. Therefore, we developed a shelf-based lyophilization protocol that effectively preserved the MSC-SP without compromising its structural or functional properties. We tested several different formulations of lyoprotectant agents, including sucrose, mannitol, and trehalose, to protect the EV membrane from freeze-drying-induced damage by encasing the EV and protein constituents within a shell made by hydrogen bonds [38]. Indeed, the EV and protein counts of the lyophilized MSC-SP were comparable to their fresh MSC-SP counterparts. Therefore, our optimized shelf-based lyophilization protocol represents an attractive method to greatly extend the shelf-life of the MSC-SP in a cost-effective manner.

The use of sugar-based excipients as protective agents has been explored during the preservation of artificial or biologically derived nanovesicles [39]. Disaccharides, such as sucrose, mannitol and trehalose, are frequently used sugar-based excipients for the cyro- and lyo-preservation of materials [40]. There are several potential mechanisms by which these sugar-based excipients can preserve the structural integrity of cryo- and lyopreserved materials. Their high glass transition temperatures could allow these excipients to maintain their amorphous structures at high temperatures, like those required for primary drying. This reduces ice crystal formation, which is a source of cryo-induced damage. Alternatively, sugar excipients could form hydrogen bonds with the biological material which prevents intracellular water loss and enables the material to retain its native conformation [40]. The stressors associated with freezing and primary drying are responsible for the loss of membrane integrity, leakage of bioactive cargo and the degradation of proteins [41,42]. We showed that the addition of disaccharide excipients greatly mitigated the influence of these stressors, with mannitol being the most effective in preserving the recovery of the EV and protein constituents of the MSC-SP.

In this study, we assessed the effectiveness of three disaccharide excipients in two lyophilization protocols. With the manifold protocol, rapid temperature reductions could pose significant stressors to the EV and protein constituents. Following manifold freeze drying, the MSC-SP formulated with trehalose had the highest protein recovery and had an EV recovery that was similar to the other SP formulations. Compared to sucrose, trehalose may have a greater ability to disrupt ice crystal formation, and may be a more effective lyoprotectant [43]. Conversely, high levels of protein and EV recovery were observed in all three formulations of MSC-SP prepared with the shelf protocol. The mannitol-formulated SP exhibited the highest EV and protein recoveries. Unlike the manifold protocol, the shelf protocol enables a careful control of the temperature and pressure conditions during each step. As a bulking agent, mannitol may mitigate the mechanical stress associated with sublimation-induced dehydration [44].

## 5. Conclusions

We have developed a method to prepare an off-the-shelf MSC-SP-based acellular therapeutic with an extended biological shelf life. TFF was used to isolate the protein and EV constituents from MSC culture supernatant in a scalable manner. Trehalose was the most effective at preserving the structural integrity of the MSC-SP following manifold-based lyophilization. This formulation of lyophilized MSC-SP also retained biological activity in vitro. A shelf protocol was developed to further improve the recovery of the freeze-dried MSC-SP constituents, with the highest recovery observed using mannitol. Thus, the appropriate selection of lyophilization protocols and protectants can be used to preserve the structural and functional integrity of MSC-SP, and thereby can address a major obstacle to developing MSC-based acellular therapeutics for clinical applications.

## Figures and Tables

**Table 1:** Thermal treatment steps for shelf-based lyophilization protocol.

Phase	Step	Temperature (°C)	Time (mins)	Ramp or Hold (R/H)	Pressure (mTorr)
Thermal treatment	1	+5	45	R	760 × 10^3^
	2	−10	35	R	760 × 10^3^
	3	−25	35	R	760 × 10^3^
	4	−40	35	R	760 × 10^3^
Primary drying	5	−30	120	R	200
	6	−22	60	R	200
	7	−22	360	H	200
	8	+5	120	R	200
	9	+20	120	R	200
	10	+20	120	H	200

**Table 2:** Size distribution profile of EV present in fresh and lyophilized MSC-SP samples.

Sample	Average Diameter (nm)	Mode Diameter (nm)	10% Distribution (nm)	50% Distribution (nm)	90% Distribution (nm)
Fresh (sucrose)	231.7	89.2	144.3	212.8	343.6
Lyophilized (sucrose)	218.4	149.1	134.8	196.1	339.2
Fresh (trehalose)	213.8	167.9	134.9	188.8	324.5
Fresh (trehalose)	219.2	166.4	134.8	195.2	343.5
Fresh (mannitol)	205.6	133.7	130.4	187.5	315.6
Lyophilized (mannitol)	207.4	142.1	126.4	183.3	323.1

## Data Availability

Not applicable.

## Abbreviations

aMSC, anoxia conditioned MSC; BCA, bicinchoninic acid assay; CM, conditioned media; DCFDA; 2′,7′-dichlorodihydrofluorescein diacetate; EV, extracellular vesicles; FBS, fetal bovine serum; hMSC, human MSC, GMP, good manufacturing practice; MSC, mesenchymal stem cells; nMSC, normoxia conditioned MSC; NTA, nanoparticle tracking analysis; ROS, reactive oxygen species; SP, secretome product; TBHP, tert-butyl hydrogen peroxide; TFF, tangential flow filtration.
